# Maternal and obstetric outcomes after Ex-Utero Intrapartum Treatment (EXIT): a single center experience

**DOI:** 10.1186/s12884-023-06129-9

**Published:** 2023-12-02

**Authors:** Marta Domínguez-Moreno, Ángel Chimenea, Lutgardo García-Díaz, Guillermo Antiñolo

**Affiliations:** 1 Department of Materno-Fetal Medicine, Genetics and Reproduction, Institute of Biomedicine of Seville (IBIS), Hospital Universitario Virgen del Rocio/CSIC/University of Seville, Seville, Spain; 2 Fetal, IVF and Reproduction Simulation Training Centre (FIRST), Seville, Spain; 3 Department of Surgery, University of Seville, Seville, Spain; 4 Centre for Biomedical Network Research On Rare Diseases (CIBERER), Seville, Spain

**Keywords:** Fetal surgery, Ex utero intrapartum treatment, Maternal outcomes, Neonatal outcomes

## Abstract

**Background:**

The Ex-utero Intrapartum Treatment (EXIT) is a procedure developed to manage a range of fetal conditions, aiming to ensure the maintenance of neonatal airway and preserving the feto-placental circulation. Its goal is to enhance the neonatal ability to successfully transition and adapt to postnatal life, thereby reducing perinatal morbidity and mortality. However, EXIT has been associated with a high risk of maternal complications. This paper provides an overview of the indications and characteristics of the EXIT procedure, as well as the obstetric outcomes and maternal complications.

**Methods:**

A retrospective analysis was conducted on a cohort of patients undergoing EXIT at our center between January 2007 and December 2022. Maternal outcomes, including demographic information, data related to the surgical procedure, surgical complications, and postoperative complications were analyzed. To assess the severity of the surgical complications, a modified Clavien-Dindo classification was used. Comparative analysis was performed by randomly selecting a sample from elective cesarean deliveries performed at our center.

**Results:**

A total of 34 EXIT procedures were performed. According to the modified Clavien-Dindo classification, we observed no major complications, while minor maternal complications were present in 2.94% of cases. Compared to elective cesarean deliveries (*n* = 350), there were no significant differences in terms of maternal complications, highlighting the similarity observed in the mean decrease in postoperative hemoglobin (1.15 g/dL in EXIT vs. 1.2 g/dL in elective cesarean deliveries, *p* = 0.94). In EXIT group, there was a higher rate of polyhydramnios (26.47% vs 6.59%, *p* < 0.001), as well as the need for amnioreduction (14.71% vs 0%, *p* = 0.001) and preterm delivery (32.35% vs 6.02%, *p* = 0.001). There were no cases of endometritis, post-procedural fever, or abruptio placentae following EXIT.

**Conclusions:**

EXIT can be considered a safe procedure when performed under adequate conditions, including appropriate uterine access and proper anesthetic management. In our series, EXIT procedure was not associated with a higher incidence of maternal complications when compared to elective cesarean delivery.

**Trial registration:**

Retrospectively registered.

## Background

Ex-Utero Intrapartum Treatment (EXIT) is a procedure developed for the management of a wide variety of fetal conditions diagnosed during pregnancy, aiming to impede the neonatal ability and successful adaptation to postnatal life [1]. Indications for EXIT procedure have been evolving during the last decades from cases of obstructing oropharyngeal tumors to a broader spectrum of fetal conditions [2–4]*.* This is the case of moderate or severe congenital diaphragmatic hernias which, according to recent studies, have achieved a notably high survival rate after EXIT procedure (82.76%—85.7% in EXIT-patients vs. 48.28% in non-EXIT patients) [5, 6]*.*

EXIT procedure involves accessing the uterine cavity and the amniotic sac, a critical step in this specialized technique. After a low transverse laparotomy, intraoperative sterile ultrasonography is used to precisely map the placental and fetal positions. In our Department, access is then achieved using an atraumatic Uterine Progressive Distractor, followed by the application of vascular clamps and a stapling device to minimize uterine bleeding. This comprehensive approach enhances maternal and obstetric outcomes, making it a promising technique for broader implementation in various medical institutions.

Advances in prenatal diagnosis, fetal imaging, maternal–fetal anesthesiology, and surgical technique and instrumentation have facilitated prenatal planning in situations associated with high neonatal morbidity and mortality [7–9]. In those cases, controlled access to fetal airway may transform a potentially life-threatening emergency into a controlled one while preserving the feto-placental circulation [6, 7, 9, 10]. Interventions performed during EXIT include endotracheal intubation, tracheostomy, removal of a temporary tracheal occlusive device, neonatal extracorporeal membrane oxygenation (ECMO) cannulation, mass excision, and others [2, 11–13]. These interventions may be performed consecutively or even within the same surgical procedure to promote neonatal cardiopulmonary stability [2].

In recent years, EXIT indications have expanded to address a broader range of prenatal anomalies. It has been increasingly utilized for the correction of non-cardiorespiratory fetal pathologies such as selected cases of gastroschisis [14–16]. In such cases, EXIT procedure enables the complete repositioning and primary closure of the prolapsed bowel during the fetal stage, preventing bowel expansion following neonatal breathing. This approach has shown significant improvements in neonatal outcomes, including reduced reliance on mechanical ventilation, shortened duration of parenteral nutrition, and earlier initiation of enteral feeding [14].

In fetal surgery, pregnant woman has been described as an "innocent bystander" [17], as the procedure does not provide direct medical benefit to her, while exposing her to potential risks during both the surgery and future pregnancies. Consequently, risks must be carefully assessed, and parenteral counseling performed before any procedure [10].

In this paper, we describe the indications and characteristics of EXIT procedure, evaluate the maternal outcomes associated, and compare the complications experienced by pregnant women who underwent EXIT procedure with those who underwent elective cesarean delivery at our Department.

## Methods

We conducted a descriptive study including all EXIT procedures performed in the Department of Maternal–Fetal Medicine, Genetics and Reproduction at Virgen del Rocío University Hospital (Seville, Spain), between July 2007 and June 2022. A comparative analysis was performed by selecting a sample of pregnant women who underwent elective cesarean delivery at our center between January 2019 and June 2022, using a simple randomization approach.

**Fig. 1:**
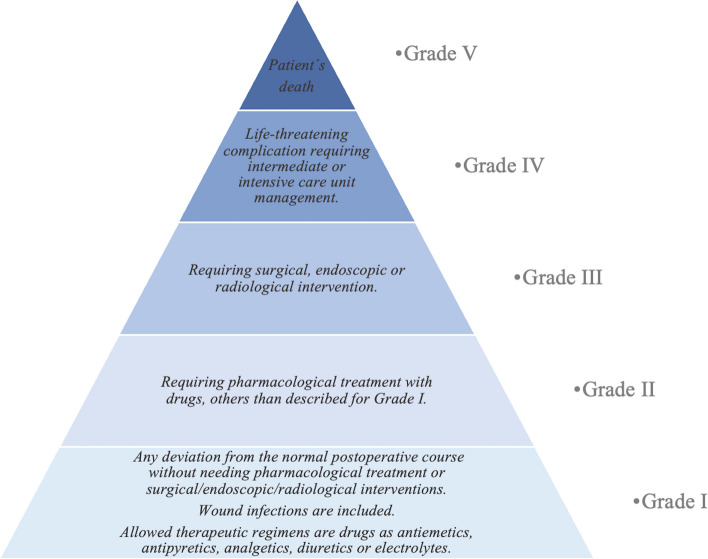
Grade complications—A modified Clavien-Dindo Classification. Aadapted from Dindo D, Demartines N, Clavien PA. Classification of surgical complications: A new proposal with evaluation in a cohort of 6336 patients and results of a survey. Ann Surg. 2004;240:205–13

In both groups, we examined maternal variables, including demographic information, data regarding the surgical procedure, surgical complications, and postoperative complications. To assess the severity of the observed complications, a modified Clavien-Dindo classification was used as a stratification tool (Fig. 1). This is a simple, reproducible, logical and comprehensive classification consisting of five severity grades used to homogenize the definition of surgical complications and to stratify them by severity [18].

Data were retrieved from the hospital’s Electronic Health Record system. Statistical analysis was performed using the IBM® SPSS Statistics 20 software package. Continuous variables are expressed as median and range. Categorial variables are expressed as frequency and percentage (%).

To compare the medians between the two groups, Student's t-test and Mann–Whitney U-test were used, depending on the normality assumption assessed by the Kolmogorov–Smirnov test. Differences in proportions were assessed using the Chi-square test, and Fisher's exact test was applied when any of the cells had a count less than five. The threshold for statistical significance was set at *p*-value < 0.05.

Informed consent was obtained from all the patients for clinical studies. Pregnant women candidates for EXIT underwent prenatal evaluation in our department, as is described elsewhere [8]. The procedure was performed by a multidisciplinary team including maternal–fetal medicine specialists, pediatric surgeons, obstetric and pediatric anesthesiologists and neonatologists.

The study has been performed in accordance with the tenets of the Declaration of Helsinki and was approved by the Institutional Ethics Review Boards from the University Hospitals Virgen del Rocío – Virgen Macarena of Seville (authorization number: 0697-N-22). Written consent for the publication of clinical data, including images, when necessary, was obtained from the patients enrolled.

## Results

**Fig. 2:**
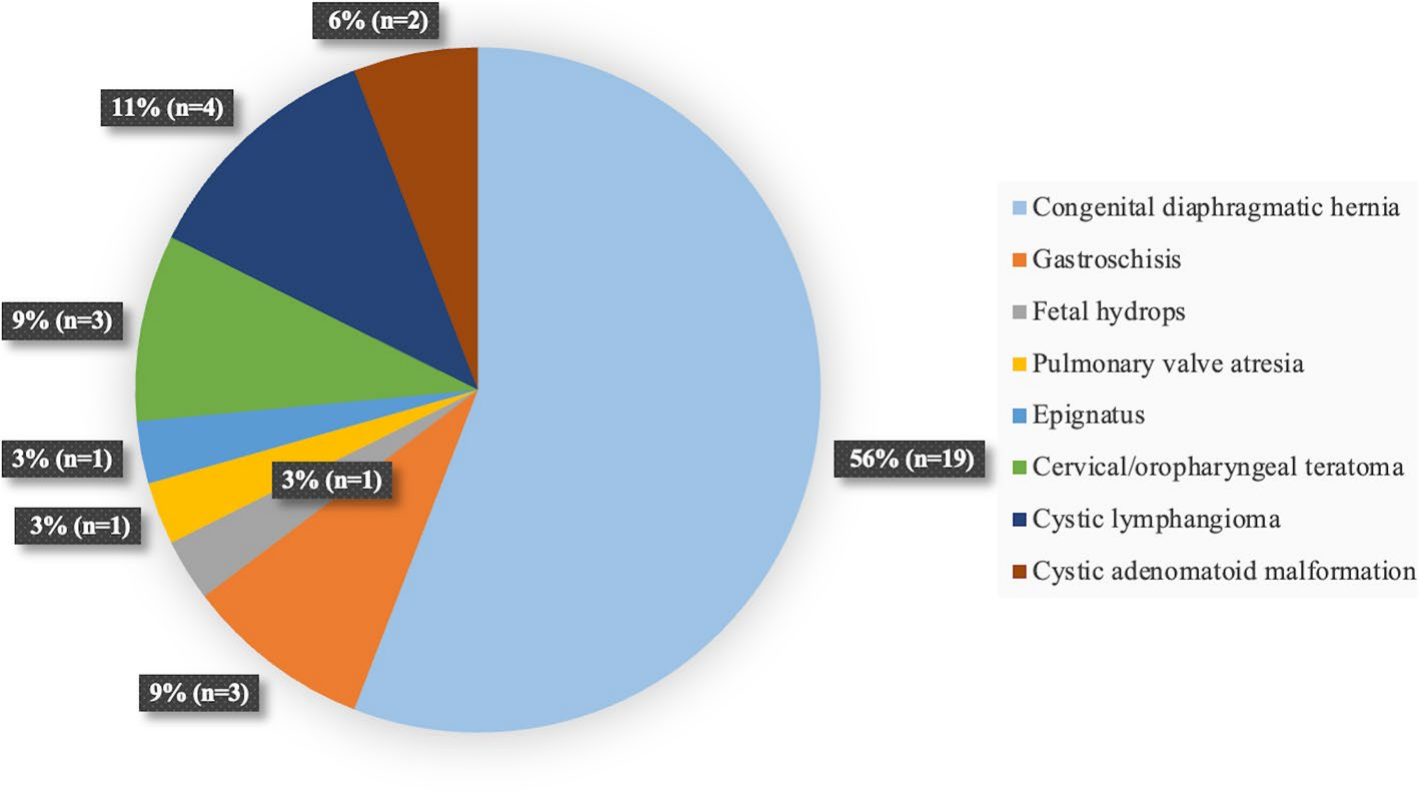
Surgical indications for EXIT

A total of 34 EXIT procedures and 350 elective cesarean deliveries performed in our center were included in the study. Indications of EXIT procedure are described in Fig. 2. The main indication was congenital diaphragmatic hernia (*n* = 19), followed by cystic lymphangioma (*n* = 4), cervical or oropharyngeal teratoma (*n* = 3) and gastroschisis (*n* = 3).

Demographic characteristics are presented in Table 1. For the EXIT group, the median maternal age was 32 years, ranging from 18 to 43 years. Of the entire cohort of pregnant women undergoing EXIT, five patients (14.7%) had a previous cesarean section. In the elective cesarean section group, the median maternal age was statistically higher (34 years, ranging from 17 to 49 years, *p*-value = 0.02), as well as a higher percentage of patients with previous cesarean Sect. (168 cases, 48.0%, *p*-value < 0.001). No significant differences were observed in obstetric pathologies such as gestational hypertension or gestational diabetes between the two groups.

**Table 1:** Demographic characteristics

**Variables**	**EXIT** **(*n* = 34)**	**Cesarean delivery (*n* = 350)**	***p*-value**
Maternal age (years), median (range)	32 (18–43)	34 (17–49)	0.018
Gravidity, median (range)	2 (1–7)	2,54 (1–11)	0.08
Parity, median (range)	0,5 (0–6)	1 (0–4)	0.39
Previous C-section, n (%)	5 (14.71)	168 (48.14)	< 0.001
Twin pregnancy, n (%)	2 (5.88)	38 (10.89)	0.56
Gestational diabetes mellitus, n (%)	0	36 (10.32)	0.06
Gestational hypertension, n (%)	1 (3.0)	31 (8.88)	0.34

Table 2 summarizes obstetric complications. The rate of polyhydramnios was higher in the EXIT group compared to the elective cesarean delivery group (26.47% vs 6.59%, *p*-value < 0.001), as well as preterm delivery (32.35% vs 6.02%, *p*-value = 0.001). No significant differences were found in the rate of preterm premature rupture of membranes between the two groups.

**Table 2:** Maternal obstetric characteristics

**Variables**	**EXIT** **(*n* = 34)**	**Cesarean delivery (*n* = 350)**	***p*-value**
Olygohidramnios, n (%)	0	4 (1.2)	> 0,99
Polyhydramnios, n (%)	9 (26.5)	23 (6.6)	< 0.001
PPROM, n (%)	2 (5.9)	6 (1.7)	0.15
Preterm delivery, n (%)	11 (32.4)	21 (6.0)	< 0.001

The surgical procedure information is summarized in Table 3. The median gestational age at the time of EXIT procedure was 37.5 weeks of gestation, which was statistically lower than observed in elective cesarean section group (39.1 weeks, *p*-value < 0.001). The median maternal and fetal operation of EXIT procedure time was 60 min and 8.5 min, respectively.

**Table 3:** Surgical procedure information

**Variables**	**EXIT (*n* = 34)**	**Cesarean delivery** **(*n* = 350)**	***p*-value**
GA at delivery (weeks), median (range)	37.4 (30.6 – 39.0)	39.0 (29.0 – 41.2)	< 0.001
Maternal length of hospital stays (days), median (range)	5 (3–58)	3 (0–36)	< 0.001
Intraoperative fever, n (%)	0	10 (2.86)	> 0.99
Pre-surgery Hemoglobin (mg/dL), median (range)	10.55 (8.2 – 10.55)	11.5 (8.1 – 15.2)	< 0.001
Post-surgery Hemoglobin (mg/dL), median (range)	9.6 (6.8 – 11.8)	10.3 (5.3 – 14)	< 0.001
Variation of Hemoglobin (mg/dl), median (range)	-1.15 (-2.9 – 0.5)	-1.2 (-6.3 – 1.8)	0.94
Percentage of Hemoglobin variation, median (range)	-10.53 (-54.31 – 18.75)	-10.9 (-28 – 4.59)	0.59
Maternal operation time (minutes), median (range)	60 (35–180)	-	-
Fetal operation time (minutes), median (range)	8.5 (3–24)	-	-

During EXIT, the median decrease in Hemoglobin was 1.15 mg/dl, compared to the decrease of 1.2 g/dl observed in the elective cesarean delivery group (*p*-value = 0.94).

Complications after surgical procedures were assessed in both groups. Notably, no cases of placental abruption were observed in the EXIT group. Postoperative complications, specifically endometritis or puerperal fever, were absent in the EXIT group. In contrast, the elective cesarean delivery group exhibited postoperative complications, with eight cases (2.3%) of puerperal fever. However, there were no statistically significant differences between the groups (*p* > 0.99). Importantly, postpartum endometritis, diagnosed clinically and characterized by leukocytosis within the range of 15,000 to 30,000 cells/microL.

Based on the former mentioned modified Clavien-Dindo classification (Table 4), there were no major maternal complications reported, and only one minor complication was recorded, namely one case of anemia requiring blood transfusion.

**Table 4:** Maternal complications after EXIT procedure. Clavien-dindo classification

**Modified Clavien-Dindo classification applied to EXIT procedure (*n* = 34)**									
**Grade I**	n	**Grade II**	n	**Grade III**	n	**Grade IV**	n	**Grade V**	n
Bleeding during/after procedure	0	Transfusion during/after procedure	1 (moderate anemia)	Severe hemorrhage requiring surgical intervention	0	Third-degree atrio-ventricular block	0	Maternal death	0
Surgical wound infection	0	Endometritis-chorioamnionitis	0	Placental abruption	0	Pulmonary thromboembolism	0		
Fever	0	Infection of another organ or system	0	Hysterectomy	0	Amniotic fluid embolism	0		
		Acute pulmonary edema	0	Uterine rupture	0	Disseminated intravascular coagulation	0		
**Total minor complications (Grade I + Grade II): 1 (2.94%)**				Bowel obstruction	0	Sepsis/ multiorgan infection	0		
				**Total major complications (Grade III + Grade IV + Grade V): 0 (0%)**					
**Total complications: 1 (2.94%)**									

After EXIT procedure, 15 women have become pregnant again so far. All of them conceived spontaneously and there were no significant obstetric complications during pregnancy. All were full term, eight of them ending by vaginal delivery and seven by cesarean section. No cases of placental accreta spectrum, bladder rupture, dehiscence or uterine rupture were identified.

## Discussion

This paper presents an analysis of obstetric and maternal outcomes following EXIT procedures conducted at our Department. In our study, there were no significant differences in terms of maternal complications between EXIT and elective cesarean deliveries. Notably, we observed a similar mean decrease in postoperative hemoglobin levels between the two groups (1.15 g/dL in EXIT vs. 1.2 g/dL in elective cesarean deliveries, *p*-value = 0.94). Furthermore, no cases of endometritis, post-procedural fever or abruptio placentae were observed following EXIT.

EXIT procedures have traditionally been associated with a high risk of maternal complications, particularly uterine hemorrhage [1, 2, 9, 11]. Maternal blood loss and the risk of hemorrhage in EXIT is usually considered to be significantly greater compared to a conventional cesarean delivery [2, 11, 19]. This increased risk can be attributed to various factors, including the duration of the procedure, the requirement for prolonged and sustained uterine relaxation, wider hysterotomy incision, and an increased likelihood of placental abruption and hysterotomy enlargement associated with EXIT [2, 10, 19, 20]. Recent data [21] have reported an average estimated blood loss of > 1,000 ml after EXIT procedure, with 6% of mothers requiring blood transfusion. Similarly, Porter et al. [19] reported an average estimated blood loss of 905 ml in a study involving 19 EXIT cases. On the contrary, we have observed no statistically significant differences in the median hemoglobin decrease in our series, when comparing EXIT (1.15 g/dL) to elective cesarean delivery (1.2 g/dL, *p*-value = 0.94). We believe that one of the main reasons is the specific surgical procedure performed at our center. Traditionally, the surgical approach for EXIT procedure has been the same as a standard lower segment cesarean Sect [10].

As described elsewhere [8], our team utilizes a different approach for the EXIT procedure. Briefly, we employ an atraumatic technique to access the uterine cavity and amniotic sac by utilizing a progressive uterine distractor. Subsequently, we placed two parallel Satinsky vascular clamps before performing the hysterotomy along the mentioned clamps using a sealing device (Premium Poly C-57 Autosuture). This unique surgical technique may contribute to the variations in outcomes compared to other reported series [2, 19, 21].

The anesthetic technique used also plays a crucial role. While epidural anesthesia is adequate for an elective cesarean delivery, the complexity of the EXIT procedure requires a well-defined anesthetic technique that involves two patients, the mother and the fetus, very different to standard obstetric general anesthesia [10, 22].

In our center, as described elsewhere [8], we use a deep general maternal anesthesia, including a general anesthesia induction (remifentanil, propofol and rocuronium) followed in rapid sequence by intubation and assisted ventilation. Before the uterine incision, deep inhalational anesthesia with sevoflurane is used to maintain uterine relaxation and preserve uteroplacental circulation and fetal gas exchange. In addition, an epidural catheter is placed to facilitate postoperative pain management of the mother. Fetal anesthesia is supplemented by an intramuscular shot (fentanyl, vecuronium and atropine) immediately after fetal exposure.

This technique provides uterine relaxation to perform EXIT appropriately. However, the requirement for potentially prolonged uterine hypotonia introduces a higher risk of uterine hemorrhage than an elective cesarean delivery.

Puerperal infection, which has been associated with longer uterine exposure, has also been reported to be increased after EXIT procedures [11, 21, 23]. It is a potentially serious complication, especially when not diagnosed and treated promptly. Previous studies have demonstrated a higher rate of surgical wound infection following EXIT compared to cesarean delivery procedures (15% vs. 2%) [23]. However, in our series, we observed no cases of wound infection. Following EXIT, none of the women experienced postoperative complications such as endometritis or post-procedural fever, in contrast to the eight cases of puerperal fever (2.29%) observed in the elective cesarean delivery group. This could be due to the systematic use of antibiotic prophylaxis (intravenous cephalosporins) in all pregnant women who underwent EXIT in our center and to the optimal postoperative care of the surgical wound on our hospital ward. In contrast, in the study described above, not all women undergoing EXIT received antibiotics. Specifically, it is reported a rate of 91% of patients, usually giving first-generation cephalosporins intravenously after the cord is clamped [23].

Another potential complication associated with EXIT procedures is uterine dehiscence or rupture in subsequent pregnancies. Abraham et al. suggested that the risk of uterine rupture in subsequent pregnancies may be higher after EXIT compared to cesarean deliveries, primarily due to the possibility of unusual sites for the hysterotomy incision [24]. While the specific risk associated with EXIT has not been extensively described, Zamora et al. reported a rate dehiscence of the former hysterotomy site of 8% in a small EXIT cohort of twenty-six pregnant women [21]. In our study, no cases of uterine dehiscence or rupture were documented in subsequent pregnancies. One of the reasons that may explain these differences could be the atraumatic access performed in our center and described above.

In addition, no significant differences were found between both groups for placental abruption (*p*-value > 0.99), placental accreta spectrum (*p*-value > 0.99) or bladder rupture (*p*-value > 0.99). Notably, none of these complications were identified after EXIT.

The main strength of our study lies in the uniformity in patient care and follow-up conditions. Pregnant women were referred from the same geographical area and all had similar demographic characteristics. Moreover, the study encompassed a significant cohort of pregnant women who underwent EXIT.

However, it is important to acknowledge the limitations of our study. The retrospective design is a significant weakness, as it may introduce potential biases and limitations in data collection. Additionally, the sample size is limited, and being a single-center study, the generalizability of the findings may be constrained. Nonetheless, it is worth noting that this study represents one of the largest series of EXIT procedures reported from a single center.

Therefore, to establish more robust conclusions, further investigation is warranted, particularly through a large-sample, multicenter study employing the same procedural approach. Conducting such research will not only help to verify the advantages of the EXIT procedure but also to improve our understanding of its safety profile and ultimately improve patient care.

## Conclusions

Although there is a perception that EXIT procedures entail a high surgical risk for mothers, our data offer compelling evidence in favor of the procedure's safety when conducted under appropriate conditions. These conditions encompass ensuring correct uterine access and employing suitable anesthetic techniques. Our findings indicate that by attentively addressing these factors, the maternal surgical risk associated with EXIT can be significantly reduced.

## Data Availability

The datasets used during the current study are available from the corresponding author on reasonable request.

## Abbreviations

EXIT: Ex-Utero Intrapartum Treatment;
ECMO: Extracorporeal membrane oxygenation
